# Abandoning the Isochore Theory Can Help Explain Genome Compositional Organization in Fish

**DOI:** 10.3390/ijms241713167

**Published:** 2023-08-24

**Authors:** Marta Vohnoutová, Anastázie Sedláková, Radka Symonová

**Affiliations:** 1 Department of Computer Science, Faculty of Science, University of South Bohemia, Branišovská 1760, 370-05 České Budějovice, Czech Republic; mvohnoutova@prf.jcu.cz; 2 Faculty of Science, University of Hradec Králové, Hradecká 1285, 500-03 Hradec Králové, Czech Republic; anastazie@sedlakovi.org; 3 Institute of Hydrobiology, Biology Centre, Czech Academy of Sciences, Na Sádkách 7, 370-05 České Budějovice, Czech Republic

**Keywords:** AT/GC genome composition, natural breaks, GC-content evolution transposons

## Abstract

The organization of the genome nucleotide (AT/GC) composition in vertebrates remains poorly understood despite the numerous genome assemblies available. Particularly, the origin of the AT/GC heterogeneity in amniotes, in comparison to the homogeneity in anamniotes, is controversial. Recently, several exceptions to this dichotomy were confirmed in an ancient fish lineage with mammalian AT/GC heterogeneity. Hence, our current knowledge necessitates a reevaluation considering this fact and utilizing newly available data and tools. We analyzed fish genomes in silico with as low user input as possible to compare previous approaches to assessing genome composition. Our results revealed a disparity between previously used plots of GC% and histograms representing the authentic distribution of GC% values in genomes. Previous plots heavily reduced the range of GC% values in fish to comply with the alleged AT/GC homogeneity and AT-richness of their genomes. We illustrate how the selected sequence size influences the clustering of GC% values. Previous approaches that disregarded chromosome and genome sizes, which are about three times smaller in fish than in mammals, distorted their results and contributed to the persisting confusion about fish genome composition. Chromosome size and their transposons may drive the AT/GC heterogeneity apparent on mammalian chromosomes, whereas far less in fishes.

## 1. Introduction

Eukaryotic genomes are compositionally heterogeneous, with a substantial global, as well as regional, variation in the ratio of adenine (A) + thymine (A) to guanine (G) + cytosine (C), called the GC-content [1,2,3]. The GC-content or GC% became an established measure of genome assemblies, although the majority of genomes are actually AT-rich (the GC% of the human genome is 40.8%) [4]. On the other hand, mammals and birds show an even more pronounced regional variation in their genome composition, where AT-rich DNA regions alternate with GC-rich ones. This is apparent as a banding pattern on their chromosomes when viewed with a special cytogenetic staining procedure [5]. The field of GC biology, which aims to explain how and why this AT/GC heterogeneity in birds and mammals arose, was largely impacted by observations of long (>200–300 kb), compositionally “fairly homogeneous” regions in mammalian genomes termed “isochores” [6,7,8]. The isochore theory shaped GC biology for decades and created a concept of “isochore families” that were arbitrarily divided into AT-rich “light” families (L1 and L2) and GC-rich “heavy” families (H1, H2, H3). For mammalian genomes, all five isochore families were reported [9,10]. For fishes, fewer and only adjacent isochore families were evidenced: e.g., the zebrafish genome consisted of two adjacent families with the lowest GC%, i.e., L1, L2; the medaka genome consisted of families L2 and H1, while in the stickleback genome, H1 and H2 occurred as in the pufferfish genome [11]. A similarly narrow range of isofamilies as for fish was reported by the same team around Bernardi for invertebrates [12]. For the chicken genome, a novel GC-rich family, H4, was introduced [13]. The concept of isofamilies was based on earlier Cs_2_SO_4_−Ag^+^ or CsCl density gradient centrifugations of random fractions of genomic DNA and observed separation of the DNA molecules by Bernardi’s team [8,14]. At that time, some short, highly repetitive regions in the human DNA were considered to produce such a pattern rather than specific genomic fractions [15,16]. As more genome assemblies became available, the concept that the mammalian genome consists of long homogeneous regions that differ in their GC% has been repeatedly disproved. In this special issue, Graur reviewed the situation [17] and demonstrated that isochores can be identified in simulated data and in a text from Moby-Dick (converted to DNA) [17]. Among earlier instances of this criticism, Cohen et al. showed that genomic regions corresponding to isochores represent only 41% of the human genome, whereby the model of isofamilies was inconsistent with their data [18]. Further, Elhaik et al. showed that the GC% at the third-codon positions (GC3) in protein-coding genes comprising about 1% of the human genome cannot be used as a proxy for the regional genomic GC% and for isochores ([19,20]). Elhaik et al. then compared segmentation algorithms designed to identify homogeneous domains as an alternative to isochores or isochoric domains. The tool identifying isochores and used by their proponents, isoSegmenter [9], requires user input to quantify sequence homogeneity and, hence, may bias the results. Elhaik et al. [2] developed IsoPlotter, an unbiased tool without user input. IsoPlotter employs a dynamic threshold computed from the length and GC% of the candidate subsequences to evaluate sequence homogeneity [19]. Applying IsoPlotter to mammalian and chicken genomes [1] depicted them as a mosaic of short and long compositionally homogeneous and non-homogeneous domains. The compositional domain model then successfully described genomes of bees [21], cows [22], body lice [23], and other organisms. Finally, Elhaik applied IsoPlotter to a panel of fish genomes and found no differences between mammalian and fish genomes [19].

To explain the compositional differences between anamniotes and amniotes, authors of the isochore theory coined the concept of a “compositional transition” [24,25], stating that “during evolution of reptiles towards birds and mammals, the gene-rich, moderately GC-rich isochores of the ancestors underwent a GC increase” [26]. However, a recent study showed that no such compositional transition occurred since the GC% of fish coding regions is higher than in birds and mammals [27].

Unfortunately, Bernardi and co-authors responded to publications that refuted the isochore theory by offending their opponents and devaluing their findings, e.g., [9,28,29,30,31]. On the other hand, the replying publications by Bernardi et al. redefined the term “homogeneous”, the length of isochores, and the number of isochore families, and criticized the inadequacy of the methods used to segment genomes. Bernardi´s team tried to compensate for the repeatedly listed limitations of the isochore theory with their tool, isoSegmenter [9]. IsoSegmenter employs a minimum threshold of 100 kb DNA fragments assessed, i.e., the sliding window size, which guarantees finding isochores ([17] and own observations by authors of this study). Despite all known issues related to isochores and isoSegmenter, the community adopted both the isochore theory and the controversial tool isoSegmetner (e.g., [32]).

One of the last doctrines related to the isochore theory that remained unchallenged was that the AT/GC heterogeneity of avian and mammalian genomes is an adaptation to their elevated body temperature. The “thermostability hypothesis” states that the higher GC% stabilizes the coding DNA regions and the corresponding RNAs and proteins [10]. Hence, for decades, mammals and birds were considered the only eukaryotes with the high(er) number of isofamilies in the single genome [10]. However, recently, both extant genera of a basal non-teleost ray-finned fish lineage called gars (Lepisosteiformes), *Lepisosteus* and *Atractosteus*, were found to exhibit mammalian-like compositional heterogeneity [5]. Their compositional heterogeneity was evidenced on chromosomes as an obvious banding pattern and in the DNA of sequenced gar genomes [5]. Interestingly, the sister lineage of gars, represented by a single species, bowfin (*Amia calva*), has a typical teleost-like AT/GC homogeneity, where only ribosomal genes are GC-rich on otherwise homogenously AT-rich chromosomes [33]. Although the reason why the ancient gar lineage differs from other fishes remains unknown, Bernardi and colleagues ignored this finding. Moreover, the publishing of plots with isofamilies ceased when tens of new genome assemblies became available. Therefore, it has not been verified whether fishes are truly AT/GC homogeneous, as stated for years before numerous genome assemblies became available [11]. 

To address this knowledge gap, we first compared plots of isofamilies sensu Bernardi and co-authors with simple histograms of GC% values in both extant gar genera with a high-quality genome assembly. Then, since the sequence size in isoSegmenter was arbitrarily set to 100 kb to maximize the yield of isochores, we tested the role of sequence size entering the assessment of GC% distribution in representatives of other fish lineages. Finally, we assessed the role of sequence size in the formation of clusters of GC% values corresponding to isofamilies. We show that the large sequence size (in terms of analyzed DNA fragments) used by Bernardi and others heavily reduced fish GC% values to label them artificially as AT/GC homogeneous. 

## 2. Results

### 2.1. IsoSegmenter Results Are Inconsistent with Histograms of GC% under the Same Conditions

Whereas Graur has [17] convincingly demonstrated isoSegmenter’s limitations in simulated data, the extent of the bias in real genomic data remains unaddressed. Hence, in parallel with describing the compositional organization of gars, we addressed the performance of isoSegementer [9] in other genomes so far unanalyzed by this tool.

**Figure 1:**
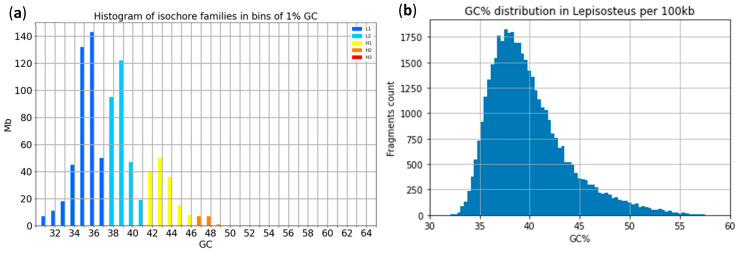
Comparison of the performance of isoSegmenter to the GC% histogram on the genome assembly of the spotted gar (*Lepisosteus oculatus*) [34]. (**a**) Graph of isochore families produced by isoSegmenter according to [9]. (**b**) A histogram of the genomic GC% using the same sliding window size of 100 kb and the same bins of 1% as in (**a**).

Applying isoSegmenter [9] with the default setting of 100 kb sliding window size to the genome assembly of the spotted gar [34], we identified four isofamilies L1, L2, H1, and H2 (Figure 1a). To compare this result, we plotted a simple histogram of the GC% values of the same genome assembly (Figure 1b) and with the same sliding window size of 100 kb. A comparison of these two plots shows that there are no peaks in the distribution of GC% values, as observed in the isoSegmenter isofamilies plot. Further, the GC% values reach almost 60% in the histogram, while they do no not reach the value of 50% in the isofamilies plot (Figure 1a,b). Finally, the low GC% values (e.g., 35%) are overrepresented in the plot of isofamilies. We note that the reference genome of spotted gar is highly incomplete when compared with the C-value of its cytological genome size [35] and that its GC% range might actually be even broader than shown here [36]. Nonetheless, these limitations cannot justify the erroneous results reported by isoSegmenter. 

Similar histograms and isofamilies plots of twelve further species are presented in the Appendix B of this study.

### 2.2. The Range of GC% Values Highly Varies with the Sliding Window Size

**Figure 2:**
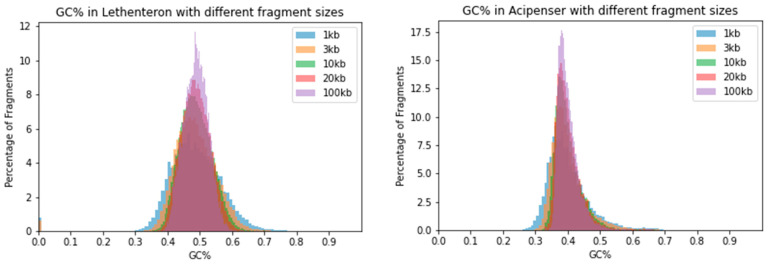
Distribution of GC% values resulting from the usage of five different sliding window sizes. Histograms of two basal non-teleost ray-finned fish species show a comparable reduction in the range of GC% values with increasing sliding window size. Left, Far Eastern brook lamprey (*Lethenteron reissneri*) of Petromyzontiformes, right sterlet sturgeon (*Acipenser ruthenus*). Note: For the histogram of GC% with the 100 kb sliding window size, which is routinely used in assessing the GC-content of genomes, the bars are too small to be viewed at the scale of the image.

To visualize the effect of the sliding window size on the distribution of GC% values, we merged histograms originating from five different window sizes into single plots in a panel of fish and fish-like species with highly diversified genomes sizes, GC% and overall genome organization sensu [37]. The selected species include the highly fragmentary and GC-rich genome of lamprey with numerous tiny chromosomes (*Lethenteron reissneri*, ca. 1 Gbp, GC = 48.74%) and an ancient polyploid genome of sturgeon (*Acipenser ruthenus,* ca. 1.8 Gbp, GC~40%) in Figure 2.

**Figure 3:**
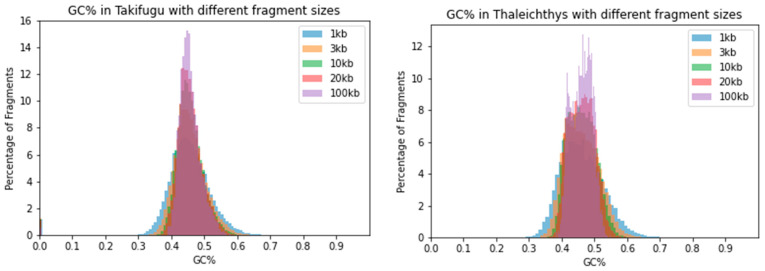
Distribution of GC% values resulting from the usage of five different sliding window sizes. Histograms of two teleost fish species show a comparable reduction in the range of GC% values with increasing sliding window size. Left, fugu (*Takifugu rubripes*) of Tetraodontiformes; right, eulachon (*Thaleichthys pacificus*) of Osmeriformes.

**Figure 4:**
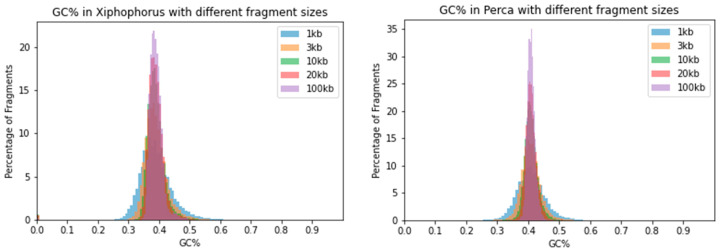
Distribution of GC% values resulting from the usage of five different sliding window sizes. Histograms of two teleost fish species show a comparable reduction in the range of GC% values with increasing sliding window size. Left, platyfish (*Xiphophorus maculatus*) of Cyprinodontiformes, and right, perch (*Perca fluviatilis*) of Perciformes.

Further, two compact teleost genomes of eulachon (*Thaleichthys pacificus*, ca. 0.5 Gbp, GC = 46.1%) and fugu (*Takifugu rubripes,* ca. 0.4 Gbp, GC = 45.7%; Figure 3). Two representatives of the standard “teleost“ genome size that is around 1 Gbp, i.e., platyfish (*Xiphophorus maculatus*, GC = 39%) and perch (*Perca fluviatilis*, GC = 40.9%; Figure 4). Plots of all species demonstrate that the window size of 100 kb highly underestimates the range of GC% values. Histograms of more species are available in the Appendix B of this study.

### 2.3. The Number of Natural GC% Clusters Varies with the Sliding Window Sequence Size

**Figure 5:**
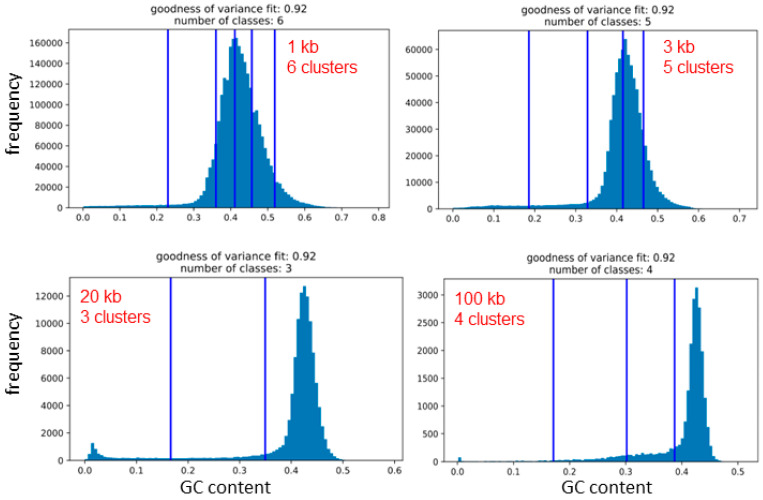
Comparison of clustering of the GC% values with four different sizes of sequences (1 kb, 3 kb, 20 kb, and 100 kb, i.e., the sliding window sizes) that each yielded a different number of GC% clusters (potentially corresponding to the number of isofamilies) delimited by blue vertical bars, the Fisher–Jenks natural breaks for the Atlantic salmon (*Salmo salar*). The y-axis represents the read frequency, and the x-axis their GC%. Note that the x-axes are not equidistant on these plots.

There are established statistical methods for clustering data into natural clusters (or groups), i.e., without any user input. One of them is the Fisher–Jenks breaks method that enables comparison plots of isofamilies (i.e., arbitrarily determined clusters of GC% values) with the resulting natural clusters of GC% values between the natural breaks. Here, we scanned the entire genome of twelve species with five different non-overlapping sliding window sizes 1 kb, 3 kb, 10 kb, 20 kb, and 100 kb (Table 1). Then, we calculated the GC% for each sequence and used these values for the Fisher–Jenks breaks method plotted as histograms with the natural breaks (Figure 5). The selected window size clearly governed the resulting counts of the natural breaks between GC% values. The counts of clusters corresponding to isofamilies range between three to seven in the species under study (Table 1). For single species, at least two different counts of natural clusters occur, with an exception in bowfin, where all sequence sizes resulted in five clusters. In the Atlantic salmon, four different counts of clusters were identified (3–6; Figure 5). The resulting clusters of GC% values are delimited by vertical bars representing the natural breaks (Figure 5). Each natural cluster corresponds to the range between two of these bars or between the bar and the border of the graph. The clusters have a high support of the values of the goodness of variance fit (GVF), reaching 0.92 where GVF = 1 means the perfect fit.

The graphic representation of the natural Fisher–Jenks breaks is provided in Figure 5 on the example of the Atlantic salmon, where three, four, five, and six natural clusters or classes were identified depending on the sliding window size used. Here, it is apparent that using the sequence sizes 1 and 3 kb, the natural clusters of GC values might roughly correspond to isofamilies since the main peaks are mostly covered by the clusters. With the increasing sequence size and the narrowing range of GC% values, the main peaks fall within a single natural cluster. It is also necessary to mention that according to Bernardi and colleagues, there is generally a fixed number of five isofamilies present in all vertebrates except for birds, where a sixth, the GC-richest isofamily, H3, exists. However, in fish genomes, usually, only two adjacent to these five isofamilies are present in a single species [10,11].

The natural clusters of GC% values differ when the sliding window size changes, not only in their count but also in their position, i.e., the GC% values of the intervals are unstable with the changing sequence size. Hence, the natural clusters of GC% values do not correspond to fixed GC% intervals of isochore families proposed by Bernardi and others.

**Figure 6:**
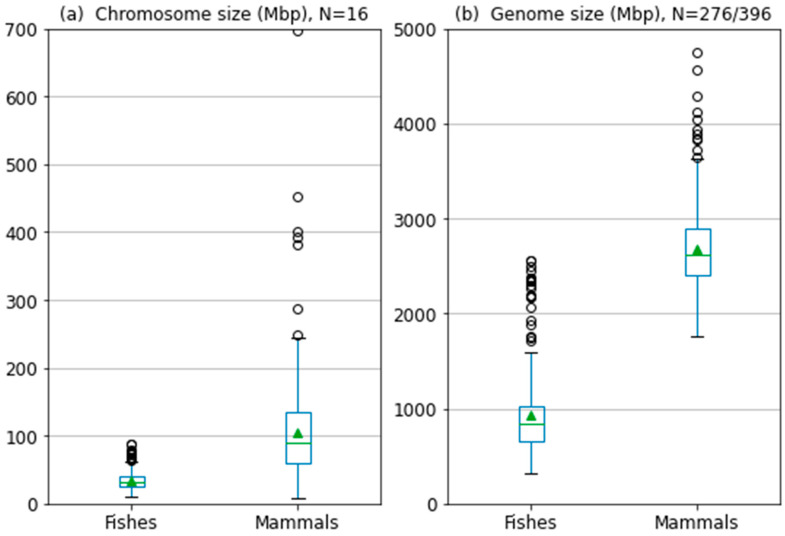
Comparison of chromosome and genome size in fish and mammals. (**a**) Chromosome size in 16 teleost fish and 16 mammalian species available at NCBI/Genome [39]. (**b**) Genome size in 276 teleost fish species and 396 mammalian species available at NCBI/Genome. Note: the outliers (circles) in teleosts are palaeopolyploid salmonids. Green triangles represent mean values.

Chromosome and genome size constitute reasons why the AT/GC heterogeneity of fish genomes was so underestimated with the setting optimal for mammals. To illustrate the striking difference between teleost fish and mammalian chromosome and genome sizes, we provide a simple comparison of genomes assembled to the chromosome level. First, sizes of single chromosomes in 16 species representing major lineages of both teleosts and mammals are plotted (Figure 6). Second, the sizes of genomes currently available at the chromosome level of both teleosts and mammals are plotted (Figure 6). The mean chromosome size is 34 Mb in teleost fish and 104 Mb in mammals. The median chromosome size is 32 Mb in teleost fish and 89 Mb in mammals. The overall genome size is roughly two and a half times higher in mammals than in teleost fishes. Despite these pronounced differences in chromosome and genome size, fish genomes have comparable GC% values [27] although they have undergone an additional whole-genome duplication [38].

## 3. Discussion

The isochore theory, i.e., the very existence of isochores, has repeatedly been falsified [1,2,17,18,31] as well as the dichotomy in the AT/GC homogeneity and heterogeneity between anamniotes and amniotes, as shown, e.g., in two gar species here and by [5]. The isochore theory stressed the higher body temperature in birds and mammals and highlighted the highest GC% values in birds with their highest body temperature [13,26]. However, the isochore theory disregarded the tiny size of avian microchromosomes, increasing the recombination rate per megabase that elevates the regional GC% [40]. A quantitative approach adding fish cytogenomics to GC biology research that was traditionally focused on birds and mammals revealed a large variability in the relationship between the chromosome size and their GC% across fish lineages [37]. Moreover, a recent study also falsified the putative “sharp increase in genic GC% during the evolution of birds and mammals” [27] proposed by Bernardi [26] along with the isochore theory. This means that a new viewpoint is needed to explore and explain the AT/GC heterogeneity in eukaryotes. The isochore theory is, however, still too deeply anchored among researchers (e.g., [40,41,42]), and it blocks any constructive discussion on the origin of the AT/GC heterogeneity by ignoring important facts (not only the aforementioned mammalian-like AT/GC heterogeneity in gars) and by actually completely omitting the alternatives. Hence, to move forward, it is necessary to cope with this barrier by interpreting the newly obtained data using novel concepts. In particular, the concept of “compositional homogeneous and non-homogeneous domains” proposed by [1,2] more suitably interprets the genome compositional organization, although without placing them in a broader biological context yet. This is important because isofamilies had been linked with crucial cellular traits involving, among others, e.g., the frequency of the CpG dinucleotides, the codon usage, and gene expression. These traits can, however, be correlated with the GC% as such without any specific need for the isochore theory. Nonetheless, there is still a clear cytogenetic difference between mammalian and avian chromosomes on one side, and fishes, amphibians, and invertebrates on the other, that needs to be understood and reconciled with in silico approaches.

### 3.1. Transposons as One of the Ways out of the Blind Alley of Isochores

There are two main groups of hypotheses trying to explain the origin of the AT/GC heterogeneity in amniotes in contrast to the AT/GC homogeneity in anamniotes (more details in [5]). These hypotheses involve (1) the selective neutral ones dominated by the GC-biased gene conversion (gBGC) emphasizing the role of chromosome size, where the increasing recombination rate (with the decreasing chromosome size) increases the GC% ([43] for review). Proponents of the gBGC mostly explore protein-coding regions (exons) that are, however, of a rather negligible genomic proportion (e.g., approx. 1.5% in the human genome [44] to about 5–10% in fish, [27]), and (2) the neo-selection hypothesis explaining GC-rich isochores as an adaptation to the increased body temperature in homeotherms [26]. However, the mammalian-like AT/GC heterogeneity in the cold-blooded ancient gars excludes both these directions because (1) according to the gBGC, the significantly smaller chromosome size should result in higher GC% values in fishes because of the stronger recombination, and (2) gars are cold-blooded vertebrates with their body temperature dependent on the environment (discussed in detail by [5]). Hence, an alternative to the two traditional groups of hypotheses was introduced—the AT/GC homogenizing effect of transposons in genomes of anamniotes [45,46]. This alternative hypothesis involves a substantially larger genome fraction in contrast to exons—the repeats and, particularly, transposons—and it utilizes the currently available tools and genome assemblies. The latest results of the bowfin genome annotation support this direction because bowfin differs in types of transposons from the AT/GC heterogenous gars [47]. Namely, bowfins show a more teleost-like transposon content dominated by DNA transposons [48] than gars. Gars have transposons that correspond more to the mammalian-like repeat composition dominated by LINE and SINE elements [34,47]. These two groups of transposons also differ in their own GC% [45]. Therefore, more research is needed on the specific GC% of the transposons involved and their distribution along chromosomes to resolve the evolutionary and mechanistic origin of the AT/GC heterogeneity, not only in gars.

### 3.2. When the Sequence Size Really Matters

Fish genomes, while being comparably GC-rich as mammals, are about two to three times smaller than mammalian ones despite the additional teleost-specific genome duplication [37]. Hence, the resolution of analytical tools may play a major role in assessing the genome compositional heterogeneity in smaller genomes with smaller chromosomes [49]. To test this, the only user input in our computational analyses was the setting of the sequence size expressed by the sliding window size used to scan the genome assembly for the GC%. This proved to have a large impact on the resulting ranges in genome GC% values. Therefore, the size-based approach of clustering DNA sequences according to their GC% is not fully suitable to tackle the AT/GC organization in eukaryote genomes. On the other hand, there is a clear difference in chromosome organization between anamniotes and amniotes, manifested as the absence of any reproducible banding pattern in fish chromosomes (with gar as the only documented exception) [5]. It still remains to explain this difference and to understand its mechanistic origin. One of the potential obstacles preventing us from explaining this difference might be the distinctly larger size of mammalian genomes and chromosomes [37]. Due to these larger sizes in mammals, the sliding window size has been traditionally set to 100 kb to produce GC% profiles of a reasonable size. In fish, such a large window size obviously hinders identifying fluctuations in the GC% [49]. There are indeed small(er)-scale fluctuations in the GC% in fish; however, they are detectable by bioinformatics only when using smaller window sizes, 1 kb or 3 kb [46]. Such fluctuations in the GC% are not detectable by cytogenetics on densely spiralized small chromosomes that sometimes do not allow for distinguishing their morphology [50,51,52]. This pattern is in line with early observations of some badly reproducible banding patterns on less spiralized chromosomes [53,54,55,56]. The insufficient cytogenetic resolution in small-sized fish chromosomes can be augmented by the homogenizing effect of repetitive elements [46] that can be highly expanded in fish chromosomes, particularly in lineages with an additional whole-genome duplication [57]. The role of genome size can also be seen in the fact that in gar and other fishes and fish-like species with a rather larger genome (e.g., bowfin, lamprey, reedfish, sturgeon), at least four isofamilies could be identified with the sliding window size 100 kb (Figure 1 and Appendix B). Whereas fishes with a smaller genome (lancelet, pike, perch, eel) show fewer isofamilies, demonstrating that genome size and the arbitrarily set window size both matter.

## 4. Materials and Methods

### 4.1. Isochore Families and GC% Histograms in the Spotted Gar (and in Other Species)

We plotted isochore families (isofamilies) in the genome assembly of two gar species [34,58] using the Python tool isoSegmenter [9]. This tool, in its default setting, utilizes the non-overlapping sliding window size of 100 kB. In parallel, we produced a simple histogram of GC% values based on the same genome assemblies with the same sliding window size as the isofamilies produced with the isoSegmenter tool. Here, the gar genome assemblies were chosen because of the already proven AT/GC heterogeneity of this peculiar ancient fish species [5]. Hence, it was highly relevant and desirable to plot isofamilies for this species. We produced histograms with the same sequence size (i.e., the sliding window size) for further fish and fish-like species listed below using our tailored Python scripts.

### 4.2. Fisher–Jenks’ Algorithm for Natural Breaks and the Goodness of Variance Fit (GVF)

Due to the observed disparity between these two ways of plotting GC% values in fish, we aimed to cluster GC% values into clusters potentially corresponding to isofamilies, however, with as little user input as possible. To do so, we applied the Fisher–Jenks optimization method for natural breaks [59,60] implemented in Python to determine the optimal number of groups/clusters of GC% values in comparison with the determination of isofamilies using the specific algorithm [9]. The Fisher–Jenks algorithm, also known as the Jenks breaks classification method, belongs to the data clustering methods and is designed to determine the best arrangement of values into different groups. Similar to the K means clustering method, the Fisher–Jenks breaks algorithm aims to partition *n* observations into *k* clusters, where each observation belongs to the cluster (group) with the nearest mean. Since this method merely clusters the values to reduce the variance within clusters and maximize the variance between clusters, it can be considered a more natural way of data clustering than the isofamilies determination by isoSegmenter. We applied this algorithm in twelve fish and fish-like chordate species (detailed below) with five different sequence sizes represented by the non-overlapping sliding window size (1 kb, 3 kb, 10 kb, 20 kb, and 100 kb) to compare the cluster numbers for each of the sequence size. 

The above-described method of clustering (of GC% values in our case) requires an a priori selection of the number of clusters or groups. This was not such an issue, as we could utilize the number of isofamilies and test them. We used the goodness of variance fit (GVF) to test the optimal number of classes. Goodness of variance (GOV) is a ratio of sum-squared deviations for cluster means (computed for each range combination) to the sum of squared deviations for array mean (computed for the entire dataset). Since GVF ranges from 1 (perfect fit) to 0 (no fit), the GVF threshold in our analysis was set to 0.9.

### 4.3. Species Studied

The natural clusters of GC% values were calculated for the following 13 fish and fish-like species, covering a broad part of basal vertebrate and ray-finned fish lineages: 1 lancelet (*Branchiostoma floridae*), 1 lamprey (*Lethenteron reissneri*), 1 skate (*Amblyraja radiata*), 1 reedfish (*Erpetoichthys calabaricus*), and 3 non-teleost ray-finned fish species, including 1 sturgeon (*Acipenser ruthenus*), 2 gar species (*Atractosteus spatula* and *Lepisosteus oculatus*), and the bowfin (*Amia calva*). Finally, five teleosts were analyzed, including one salmonid (*Salmo salar*) known for its salmonid-specific genome duplication and a high proportion of GC-rich repeats [57]. The remaining four teleosts were one eel (*Anguilla anguilla*), one platyfish (*Xiphophorus maculatus*), one perch (*Perca fluviatilis*), and one pike (*Esox lucius*). Isofamilies were plotted for the same panel of species using the isoSegmenter tool [9].

## 5. Conclusions

We show how the isochore theory and the tool designed to identify isofamilies heavily distort the real distribution of GC% values, particularly in fishes with smaller chromosomes and genomes. Hence, removing the constraints imposed by alleged isochores and isofamilies that artificially labeled fish genomes globally as AT/GC homogenous will move our understanding of vertebrate evolution toward the real background of their cytogenetic homogeneity. There might be a certain level of AT/GC genome homogenization caused by the accumulation of transposons with similar GC% values in fishes. At the same time, transposons of highly different GC% values might be contributing to the AT/GC heterogeneity in mammals and gar and, potentially, in other, so far unexplored species and lineages. Chromosome size has to be taken into account when analyzing the AT/GC composition and organization of fish genomes.

## Figures and Tables

**Table 1:** An overview of how the sequence size (1 kb, 3 kb, 10 kb, 20 kb, and 100 kb) determines the resulting count of the natural clusters of GC% values in twelve fish and fish-like species with large spans in genome size and GC% values and covering the phylogenetic tree from a lancelet to teleosts. The AT/GC heterogenous fish genome is represented by the alligator gar (*Atractosteus spatula)*.

Species	1 kb	3 kb	10 kb	20 kb	100 kb
*Acipenser ruthenus*	5	5	5	6	5
*Amblyraja radiata*	6	6	6	6	4
*Amia calva*	5	5	5	5	5
*Anguilla anguilla*	5	5	5	4	4
*Atractosteus spatula*	5	6	6	6	5
*Branchiostoma floridae*	6	6	5	5	5
*Erpetoichthys calabaricus*	6	6	6	6	5
*Esox lucius*	5	5	5	6	6
*Lethenteron reissneri*	5	6	5	5	5
*Perca fluviatilis*	6	6	7	7	6
*Salmo salar*	6	5	4	3	4
*Xiphophorus maculatus*	5	6	6	6	6

**Figure A1:**
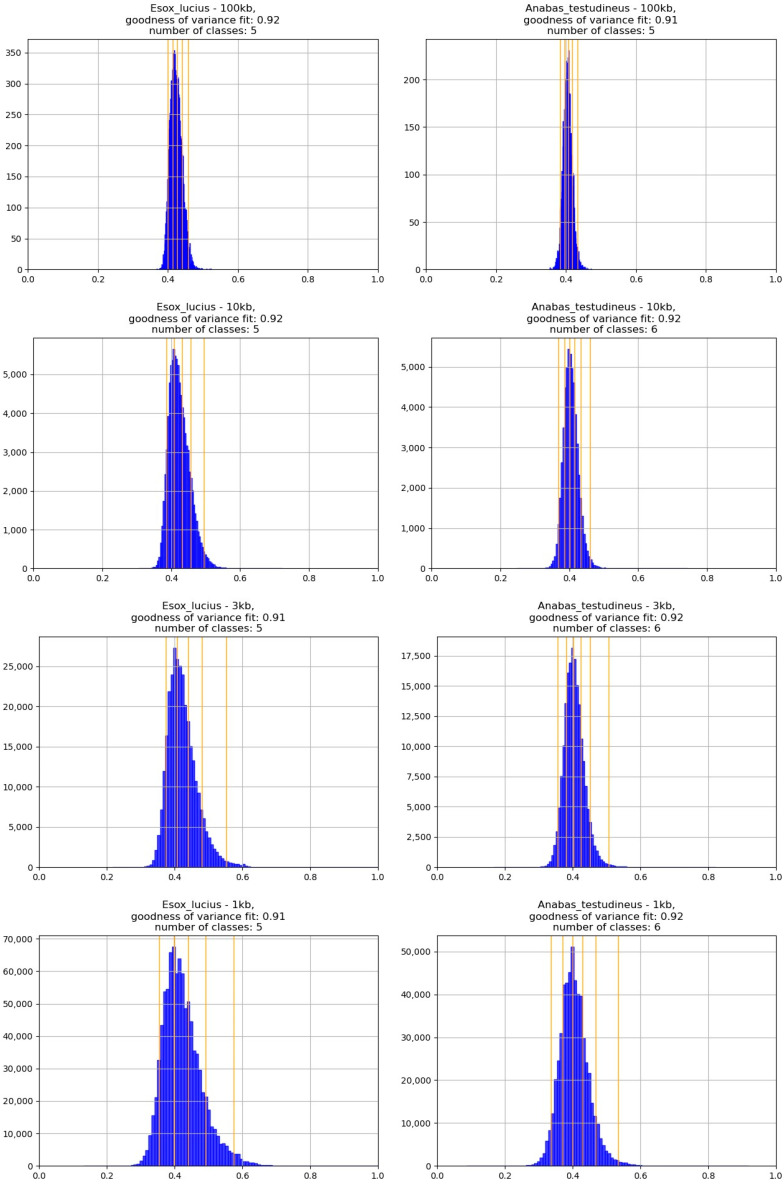
Two teleost fish species with high-quality genome assemblies that were analyzed here demonstrate the effects of the sliding window size on the range of GC% with four window sizes (100 kb, 10 kb, 3 kb, 1 kb). Left, the Northern pike; right, climbing perch.

**Figure A2:**
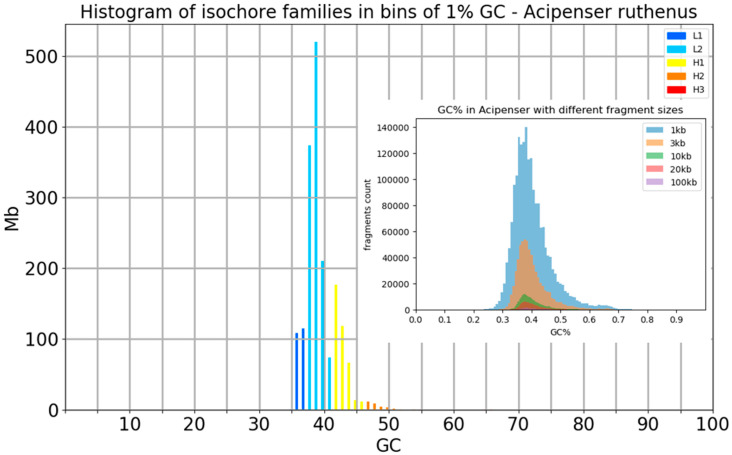
Direct comparison of the graph of isofamilies produced by isoSegmenter using the default 100 kb sliding window size [9] and histograms with five different DNA sequence sizes in 12 fish and fish-like species (see Supplementary Materials). Here, the histograms (insets) are shown with the absolute counts of sequences of the given sequence (sliding window) size on the X-axis since the plots of isofamilies are plotted in megabases. Note that in the histogram with the 100 kb sequence size, the violet color is actually invisible.

## Data Availability

Genome assemblies analyzed in this study originate from the public databases NCBI https://www.ncbi.nlm.nih.gov/genome and www.ensembl.org (accessed in September 2022). Data originating from this and down-stream studies are available in full-size format on our online project repository https://github.com/martavohnoutova/Evan.

## Supplementary Materials

The following supporting information can be downloaded at: https://www.mdpi.com/article/10.3390/ijms241713167/s1.
