# Supplementary material for: Abandoning the Isochore Theory Can Help Explain Genome Compositional Organization in Fish
Source: Int J Mol Sci. 2023 Aug 24;24(17):13167. doi: 10.3390/ijms241713167 (PMC10487504; doi:10.3390/ijms241713167)
Supplement: Supplementary file 1 [file ijms-24-13167-s001.zip › ijms-2482825-supplementary.pdf]

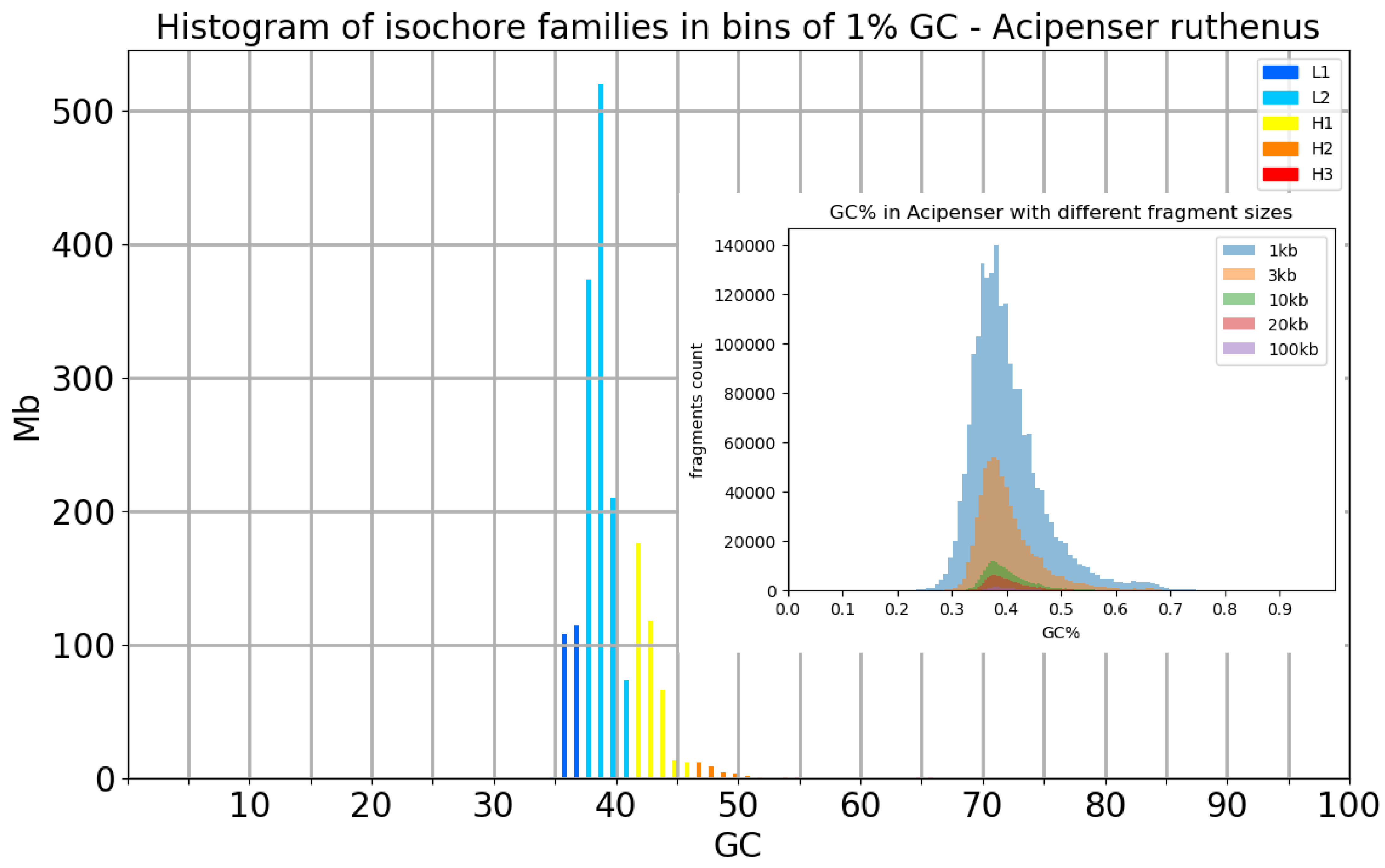
Histogram of isochore families in bins of 1% GC - Acipenser ruthenus
Mb
GC
0
100
200
300
400
500
10
20
30
40
50
60
70
80
90
100
L1
L2
H1
H2
H3
GC% in Acipenser with different fragment sizes
fragments count
GC%
0
20000
40000
60000
80000
100000
120000
140000
0.0
0.1
0.2
0.3
0.4
0.5
0.6
0.7
0.8
0.9
1kb
3kb
10kb
20kb
100kb

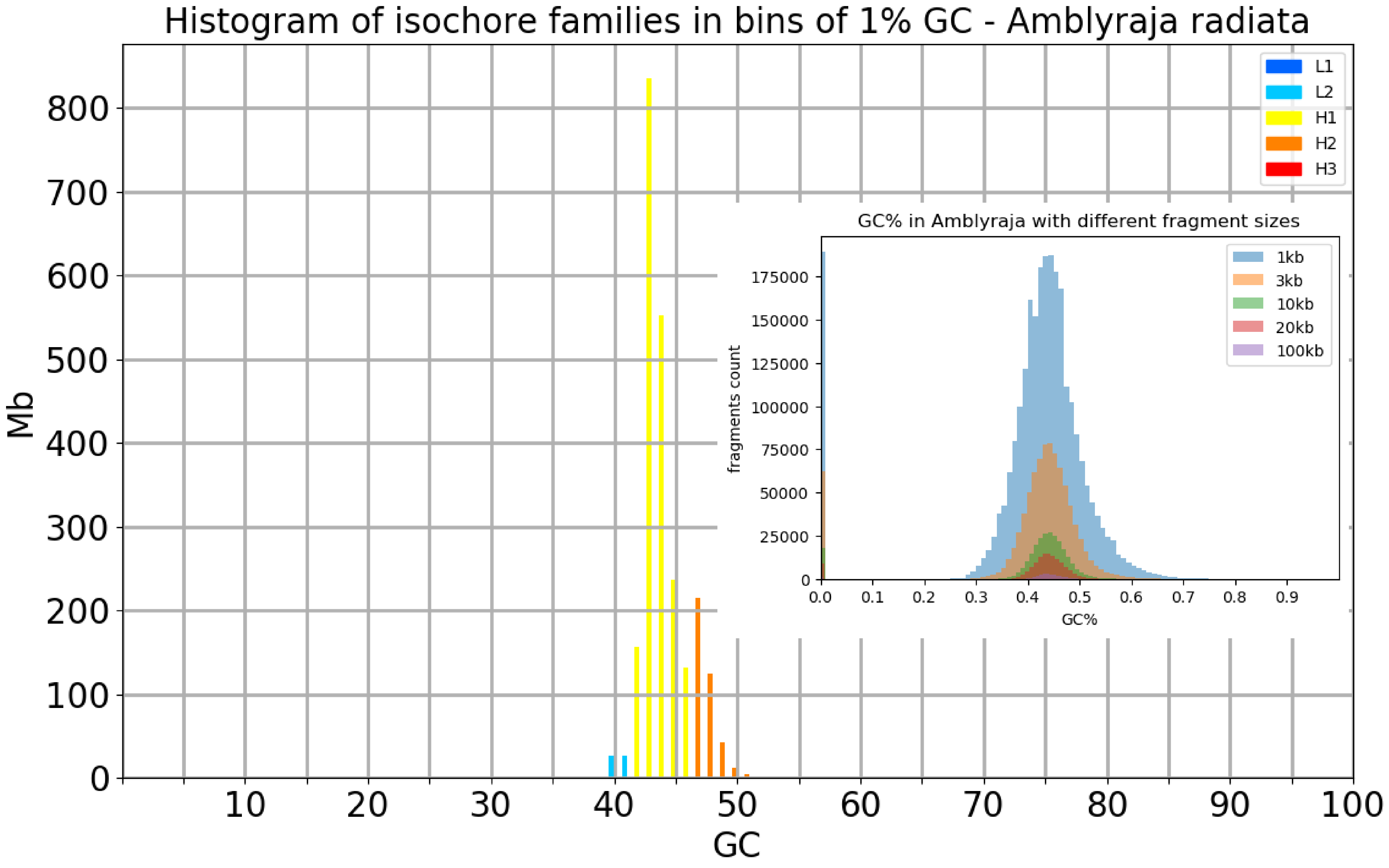

Histogram of isochore families in bins of 1% GC - Amblyraja radiata
Mb
GC
L1
L2
H1
H2
H3
GC% in Amblyraja with different fragment sizes
fragments count
GC%
1kb
3kb
10kb
20kb
100kb

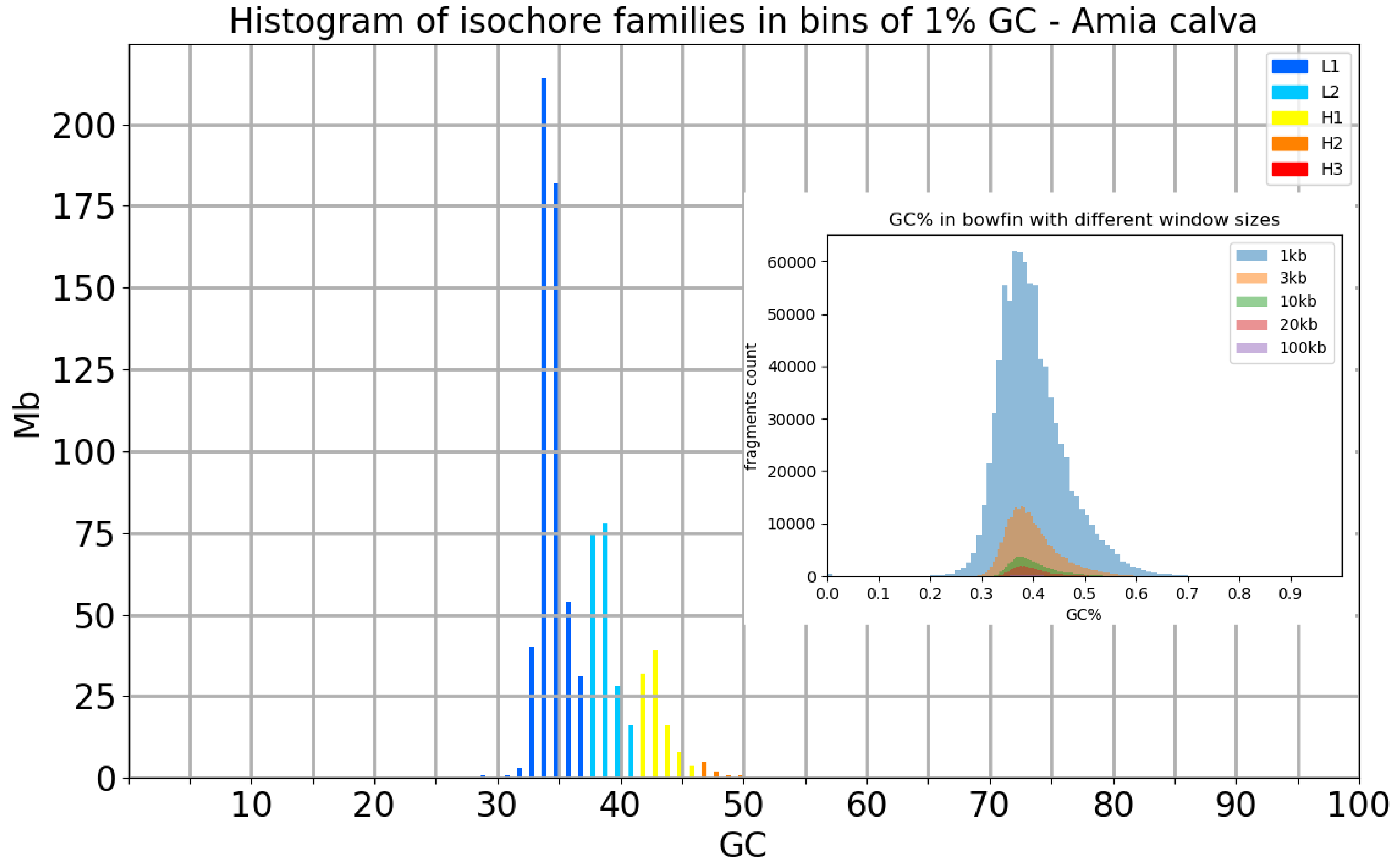

Histogram of isochore families in bins of 1% GC - Amia calva
Mb
GC
L1
L2
H1
H2
H3
GC% in bowfin with different window sizes
fragments count
GC%
1kb
3kb
10kb
20kb
100kb

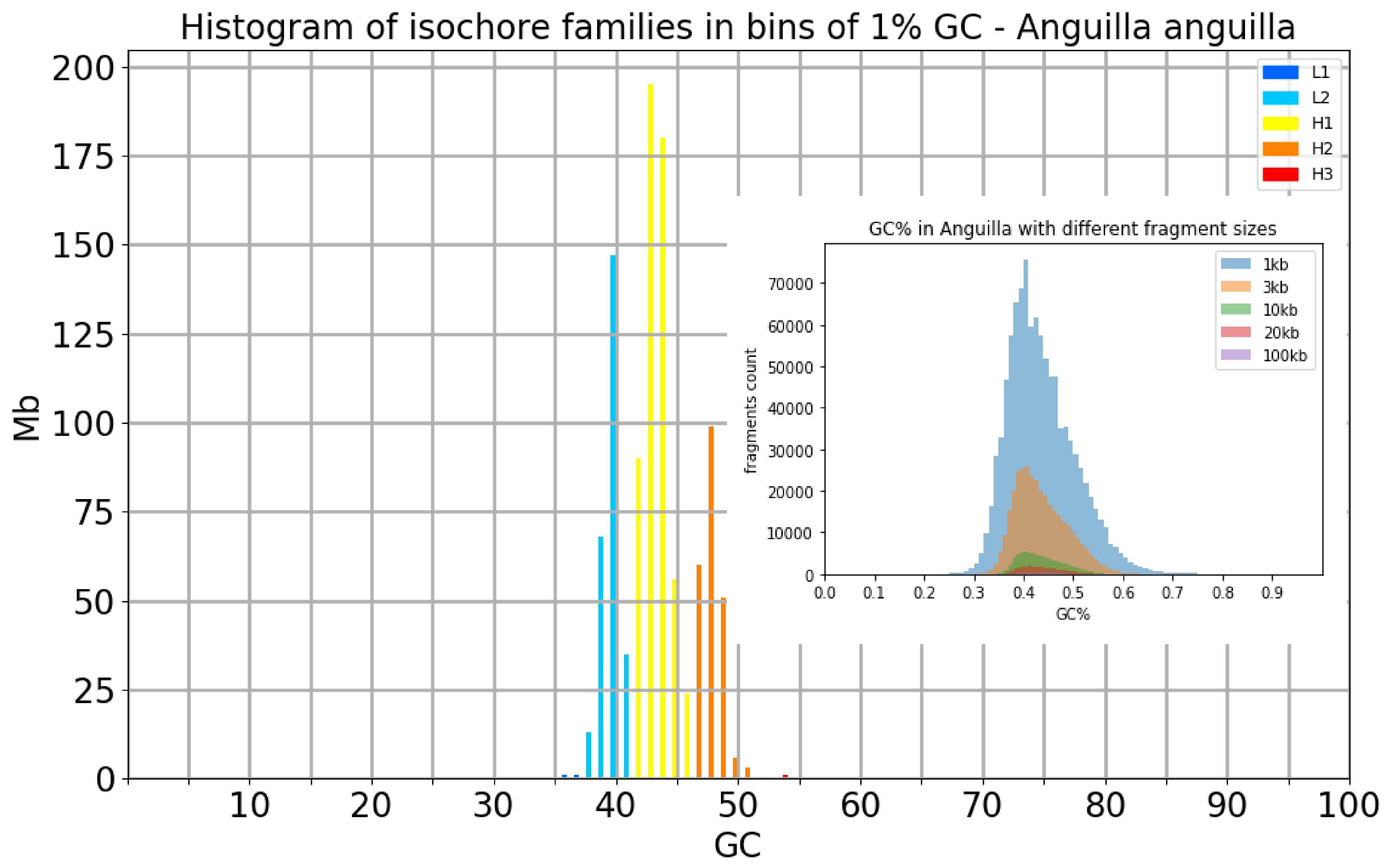
Histogram of isochore families in bins of 1% GC - Anguilla anguilla
Mb
GC
L1
L2
H1
H2
H3
GC% in Anguilla with different fragment sizes
fragments count
GC%
1kb
3kb
10kb
20kb
100kb

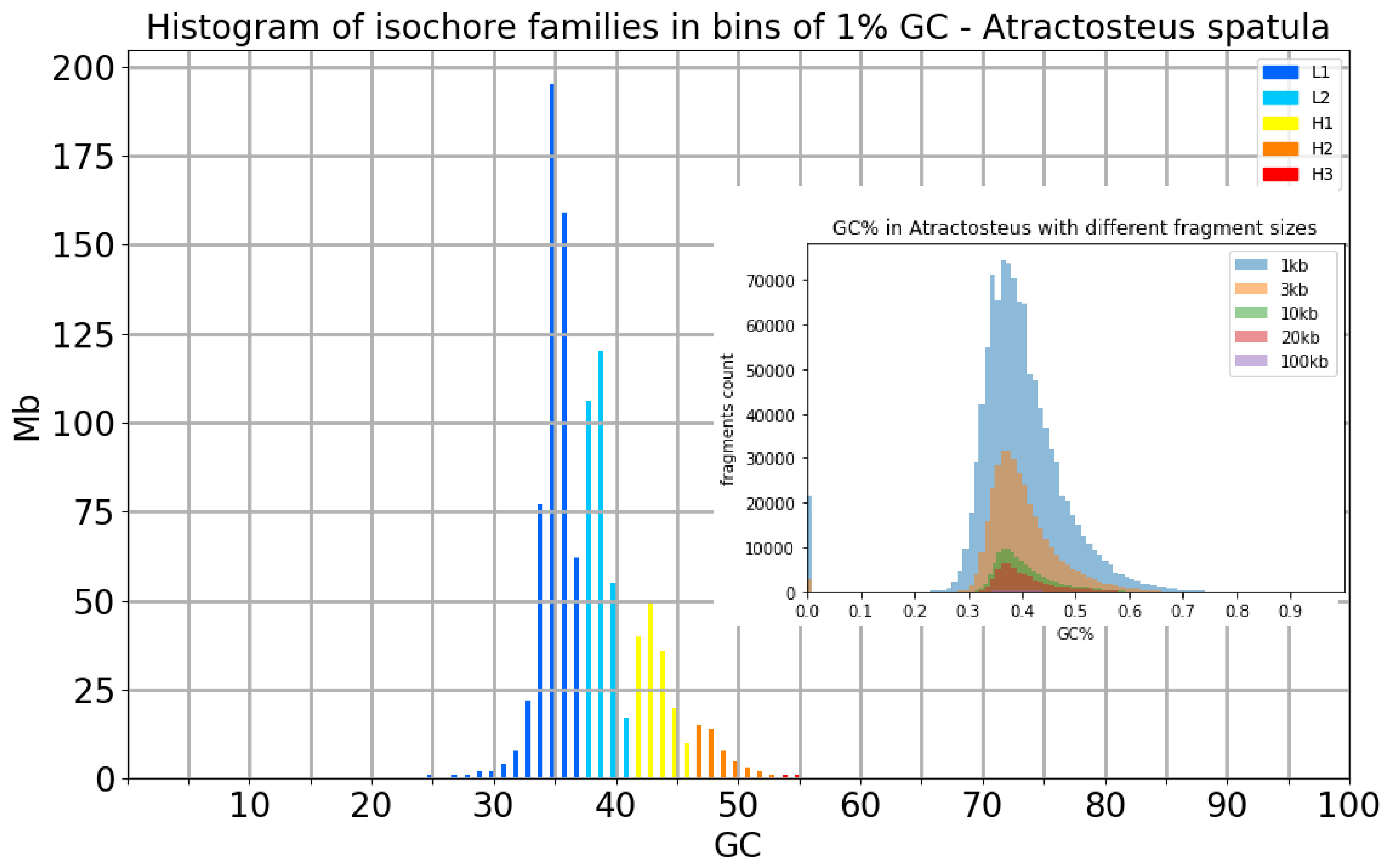
Histogram of isochore families in bins of 1% GC - Atractosteus spatula
Mb
GC
0
25
50
75
100
125
150
175
200
10
20
30
40
50
60
70
80
90
100
L1
L2
H1
H2
H3
GC% in Atractosteus with different fragment sizes
fragments count
GC%
0
10000
20000
30000
40000
50000
60000
70000
0.0
0.1
0.2
0.3
0.4
0.5
0.6
0.7
0.8
0.9
1kb
3kb
10kb
20kb
100kb

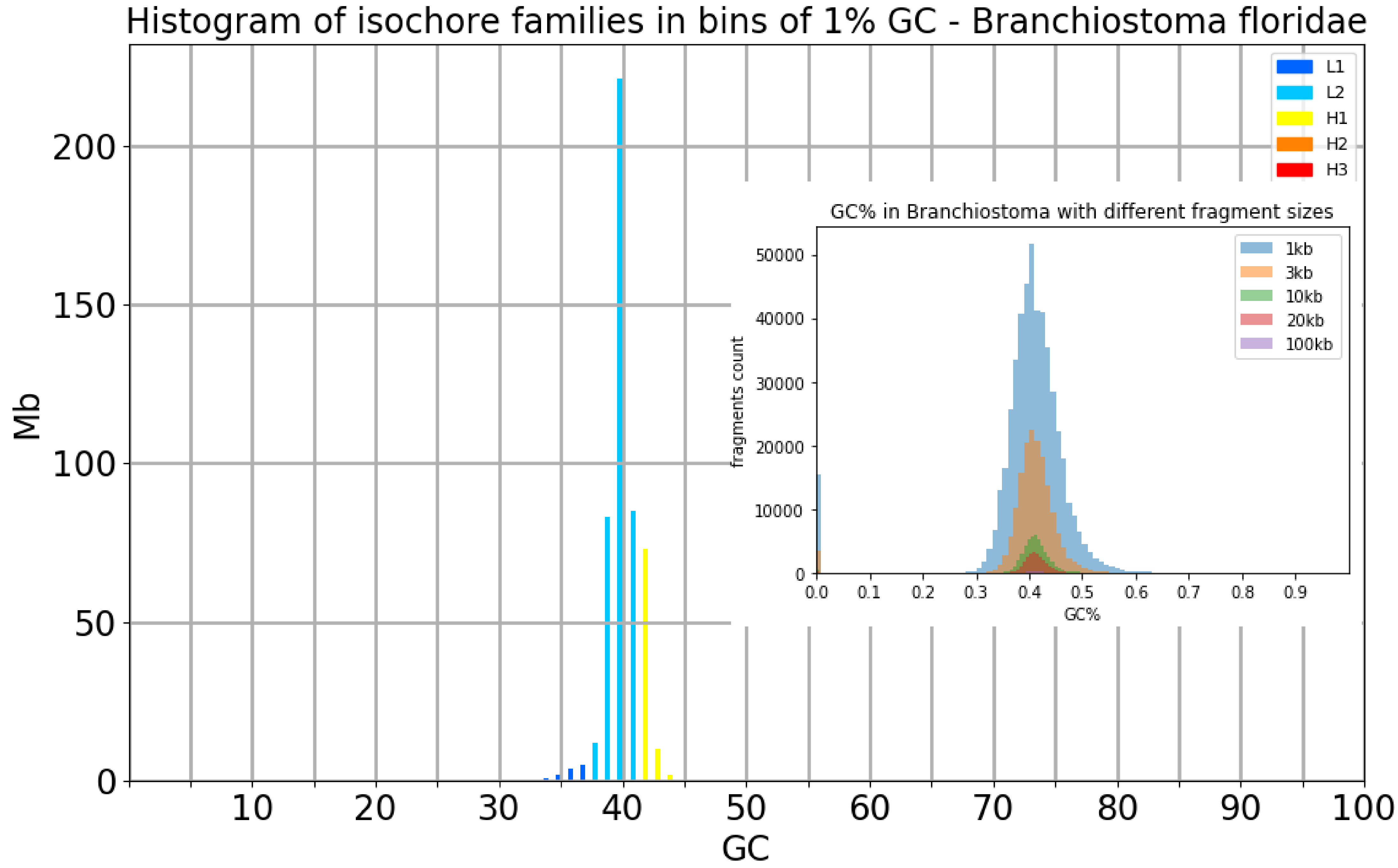

Histogram of isochore families in bins of 1% GC - Branchiostoma floridae
Mb
GC
0
50
100
150
200
10
20
30
40
50
60
70
80
90
100
L1
L2
H1
H2
H3
GC% in Branchiostoma with different fragment sizes
fragments count
GC%
0
10000
20000
30000
40000
50000
0.0
0.1
0.2
0.3
0.4
0.5
0.6
0.7
0.8
0.9
1kb
3kb
10kb
20kb
100kb

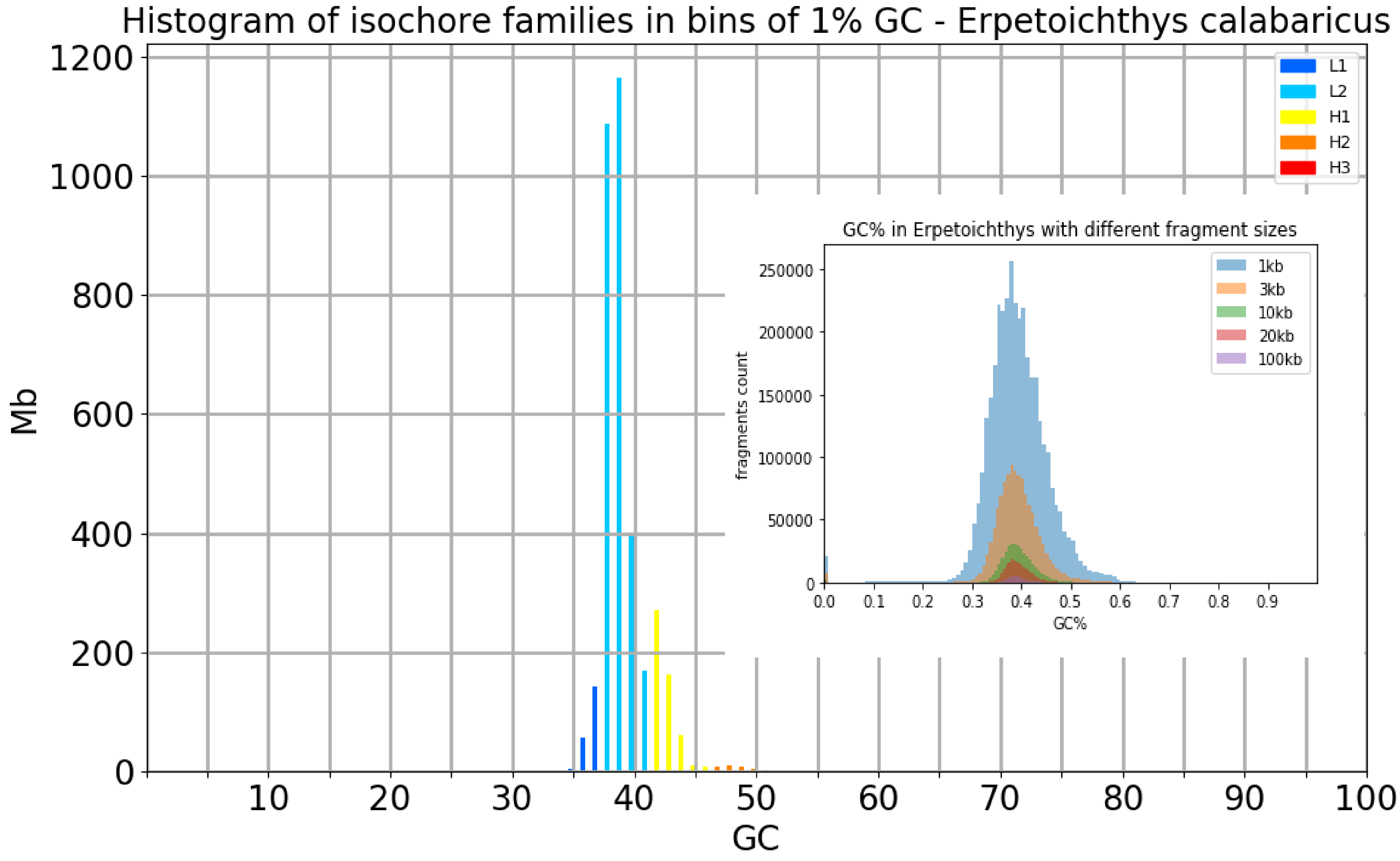
Histogram of isochore families in bins of 1% GC - Erpetoichthys calabaricus
Mb
GC
L1
L2
H1
H2
H3
0
200
400
600
800
1000
1200
10
20
30
40
50
60
70
80
90
100
GC% in Erpetoichthys with different fragment sizes
fragments count
GC%
1kb
3kb
10kb
20kb
100kb
0
50000
100000
150000
200000
250000
0.0
0.1
0.2
0.3
0.4
0.5
0.6
0.7
0.8
0.9

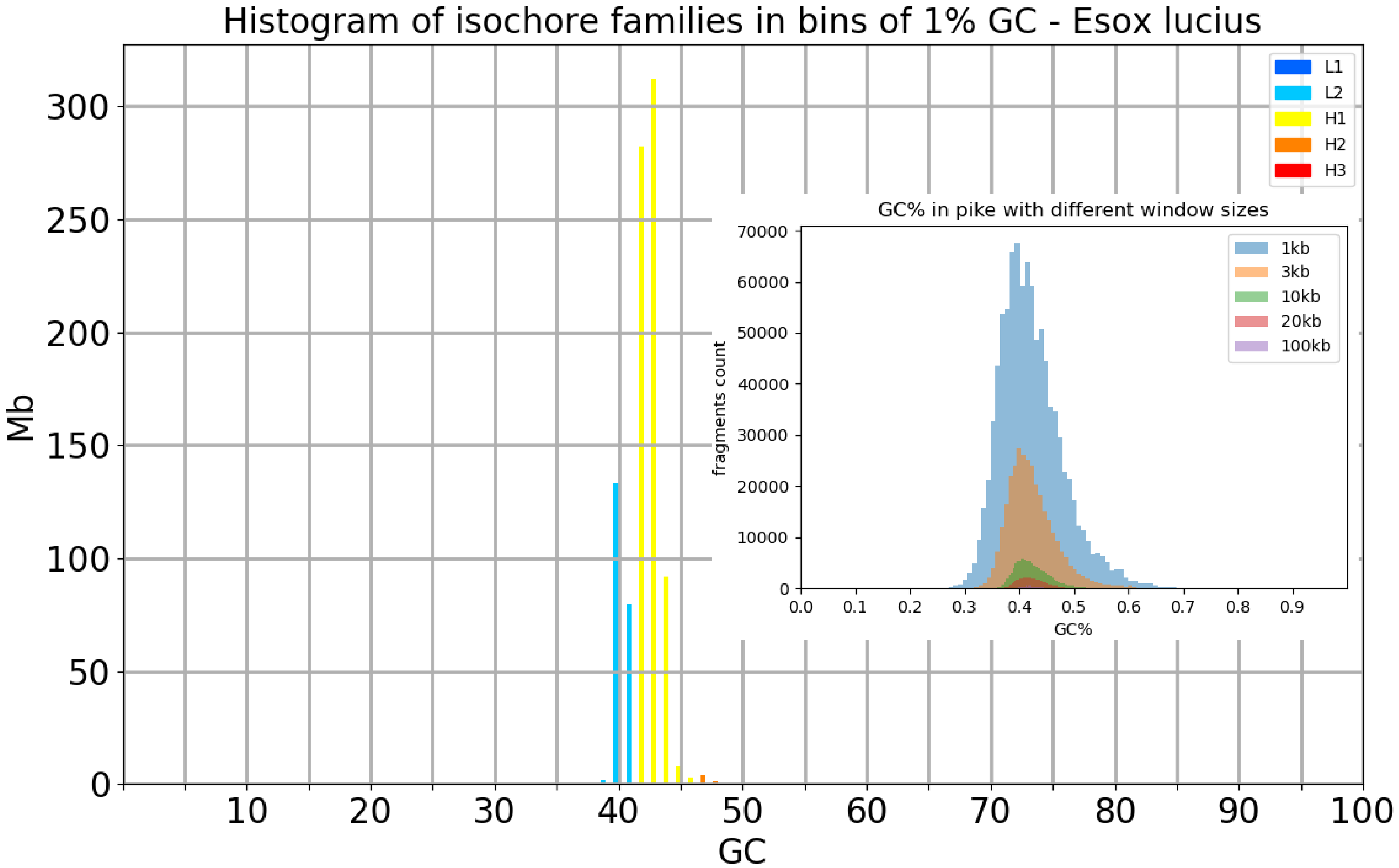

Histogram of isochore families in bins of 1% GC - Esox lucius
Mb
GC
L1
L2
H1
H2
H3
GC% in pike with different window sizes
fragments count
GC%
1kb
3kb
10kb
20kb
100kb

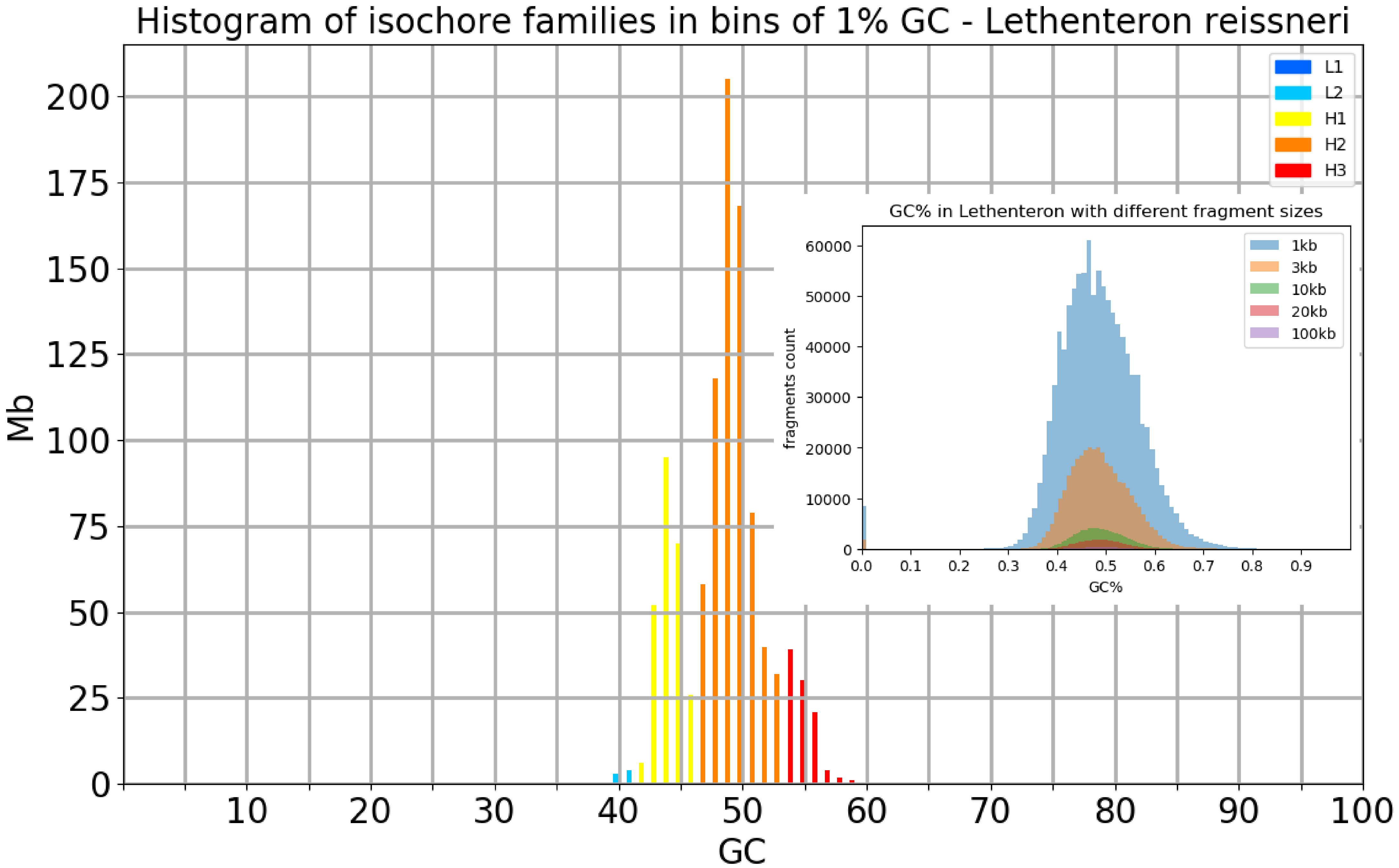

Histogram of isochore families in bins of 1% GC - Lethenteron reissneri
Mb
GC
0
25
50
75
100
125
150
175
200
10
20
30
40
50
60
70
80
90
100
L1
L2
H1
H2
H3
GC% in Lethenteron with different fragment sizes
fragments count
GC%
0
10000
20000
30000
40000
50000
60000
0.0
0.1
0.2
0.3
0.4
0.5
0.6
0.7
0.8
0.9
1kb
3kb
10kb
20kb
100kb

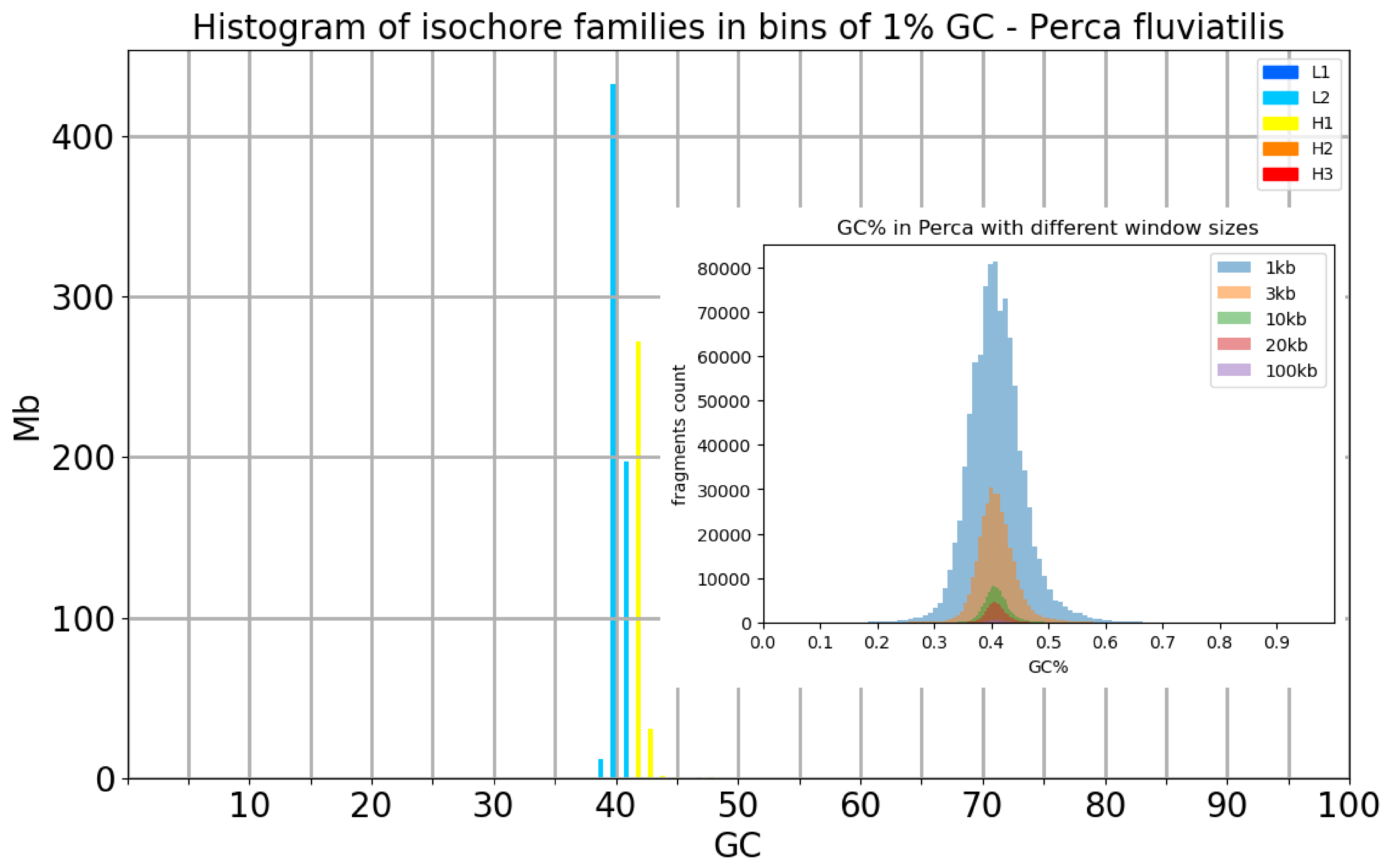

Histogram of isochore families in bins of 1% GC - Perca fluviatilis
Mb
GC
L1
L2
H1
H2
H3
GC% in Perca with different window sizes
fragments count
GC%
1kb
3kb
10kb
20kb
100kb

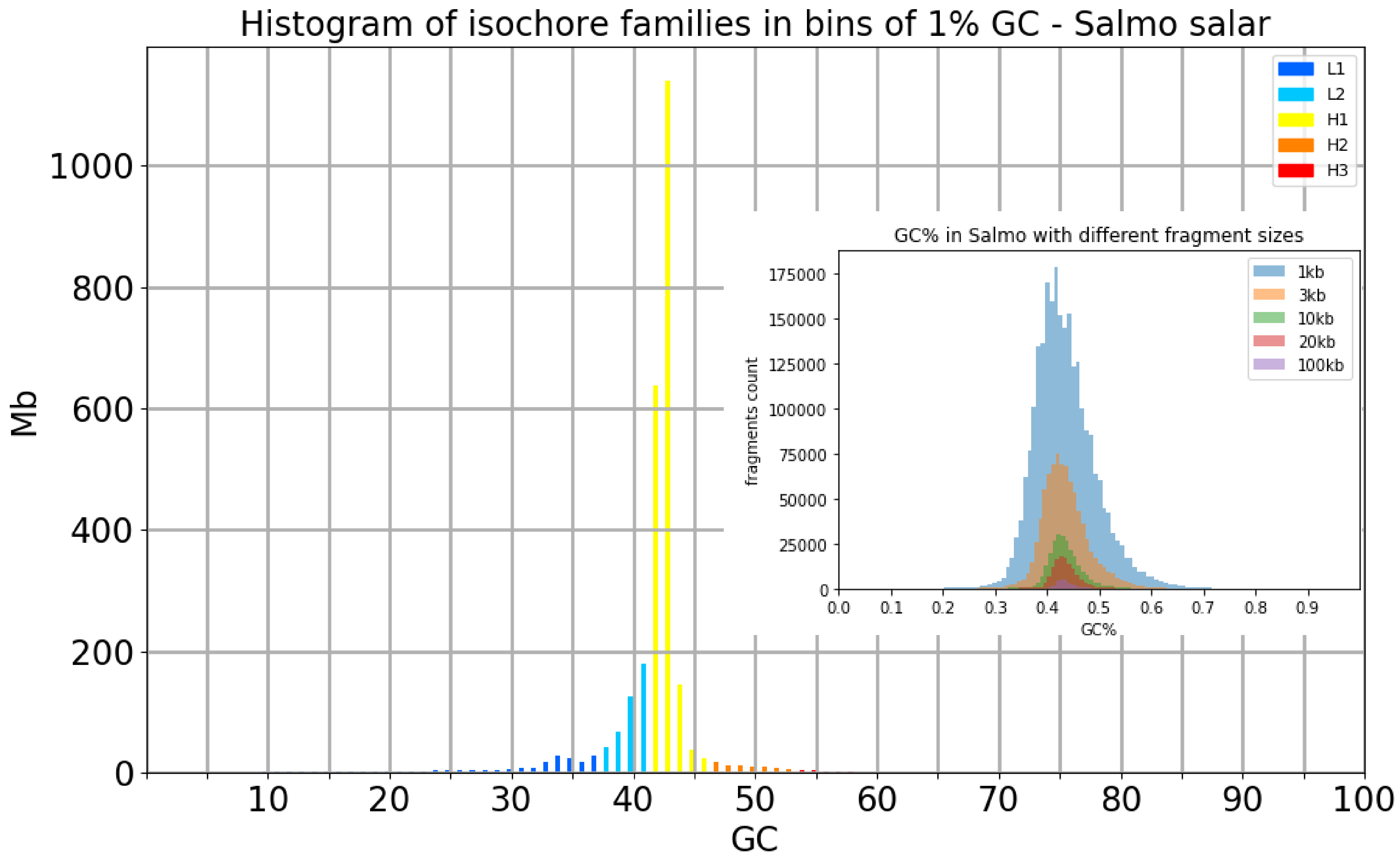
Histogram of isochore families in bins of 1% GC - Salmo salar
Mb
GC
L1
L2
H1
H2
H3
GC% in Salmo with different fragment sizes
fragments count
GC%
1kb
3kb
10kb
20kb
100kb

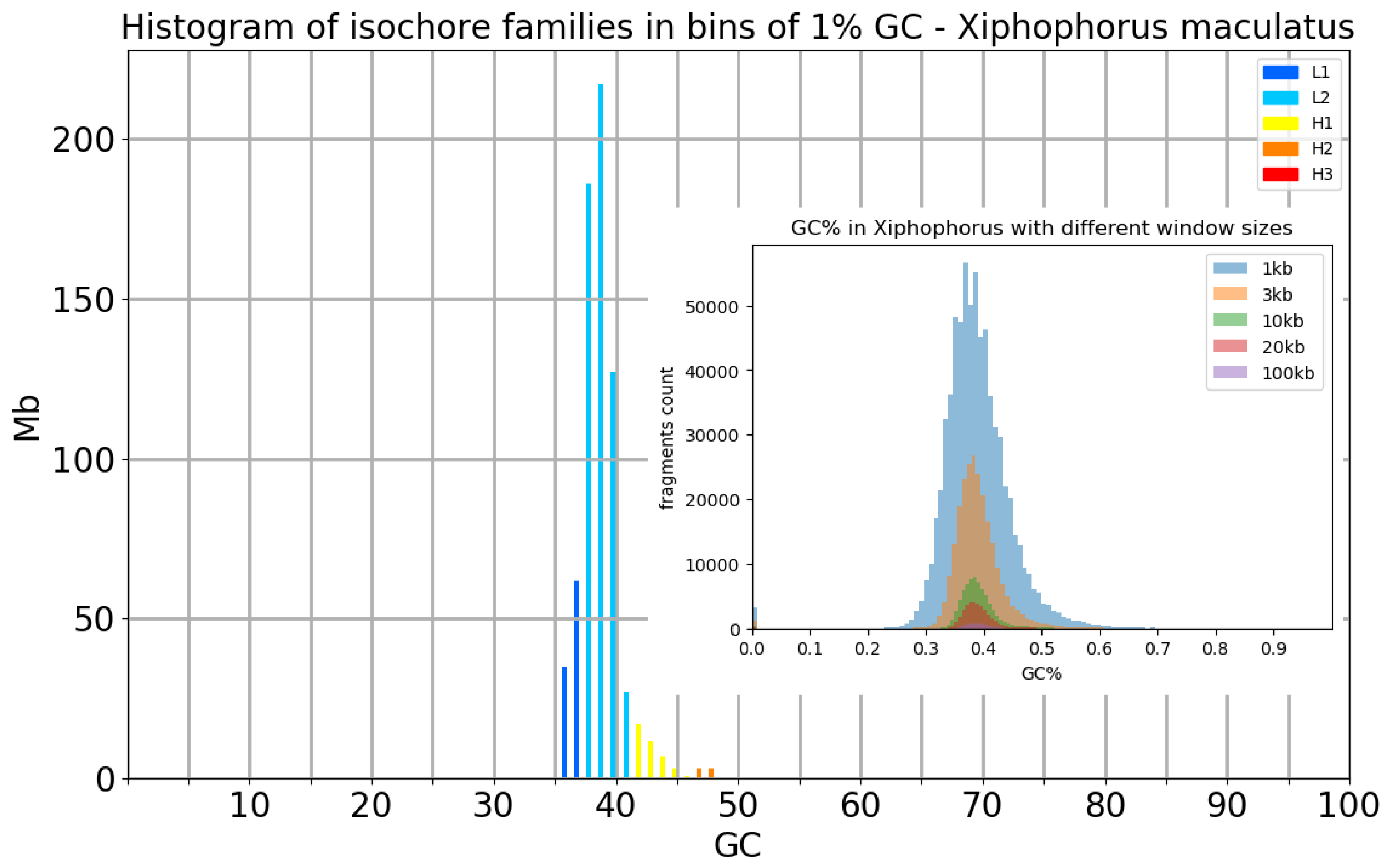
Histogram of isochore families in bins of 1% GC - Xiphophorus maculatus
Mb
GC
L1
L2
H1
H2
H3
0
50
100
150
200
10
20
30
40
50
60
70
80
90
100
GC% in Xiphophorus with different window sizes
fragments count
GC%
1kb
3kb
10kb
20kb
100kb
0
10000
20000
30000
40000
50000
0.0
0.1
0.2
0.3
0.4
0.5
0.6
0.7
0.8
0.9
